# Strange Saucer-Shaped Lung Collapse Successfully Salvaged in Spite of Delayed Intervention

**DOI:** 10.7759/cureus.67784

**Published:** 2024-08-26

**Authors:** Tushar Sahasrabudhe, Mithun Nilgiri K, Ashish R Dolas, Rishi G Orakkan

**Affiliations:** 1 Respiratory Medicine, Dr. D. Y. Patil Medical College, Hospital & Research Centre, Dr. D. Y. Patil Vidyapeeth (Deemed to be University), Pune, IND; 2 Cardiothoracic and Vascular Surgery, Dr. D. Y. Patil Medical College, Hospital & Research Centre, Dr. D. Y. Patil Vidyapeeth (Deemed to be University), Pune, IND

**Keywords:** rare shape, saucer shaped, multidisciplinary approach, bronchoscopy, orthopnea, diaphragmatic plication, thoracotomy, mitral valve replacement, diaphragmatic palsy, lung collapse

## Abstract

A middle-aged woman presented in November 2023 with exertional dyspnea and a chronic cough for three months. She had undergone a repeat mitral valve replacement (MVR) surgery five months prior. She had a tissue MVR in 2016, which degenerated, making her symptomatic, and hence had to be replaced with a metallic valve. As the respiratory symptoms recurred two months post-op, she was evaluated for the integrity of the newly placed mitral valve, which was found to be functioning well. The left ventricular function was well preserved, and she had no vegetation or clots. She was therefore investigated further for other possible causes. Chest X-ray showed a strange saucer-shaped (or disc-shaped) opacity above the middle portion of the right hemidiaphragm, which itself was found to be elevated. An ultrasound of the chest ruled out subpulmonic pleural effusion and confirmed right hemidiaphragm palsy. A computed tomography (CT) scan of the thorax was suggestive of a strange-shaped collapse of the right lower lobe with tortuous air bronchograms and a small intraluminal soft tissue shadow in the right lower lobe bronchus. A bronchoscopy confirmed collapsing segments of the right lower lobe due to external compression. It also ruled out any intrabronchial pathology causing obstruction, effectively confirming that the tissue shadow was probably just a mucus plug. A possible phrenic nerve injury during thoracotomy at the time of MVR was thus concluded. A diaphragmatic plication was advised considering that she had significant orthopnea and low peripheral oxygen saturation and that the collapsed lung would not possibly expand beyond six months or so and could in itself act as a focus for further mucus stagnation, leading to infection and further bronchiectasis, which had probably already started developing. It was difficult to persuade the patient for a third thoracotomy and she took much time to decide. Good counselling, rapport building, and assurance that, although the lung may not fully expand beyond six months, at least the orthopnea would significantly improve, she finally consented to diaphragmatic plication, which was done after 10 months of the MVR surgery. Aggressive post-operative chest physiotherapy and rehabilitation were promptly initiated. The lung completely expanded one month post-op and was thus successfully salvaged.

## Introduction

Incomplete inflation of the lung or part of it is called lung collapse [1]. Four common pathological mechanisms responsible for lung collapse are (a) obstruction of the airway, also called obstructive collapse; (b) compression of the lung by an intrathoracic, extrathoracic, or chest wall lesion, also called compressive collapse; (c) destruction of the lung causing volume loss, also called fibrotic collapse; and (d) inability of the lung to expand because of a thick layer of visceral pleura overlying it, also called adhesive collapse [2].

To diagnose lung collapse radiologically, some major and minor signs have been suggested by Dr. Benjamin Felson [3]. A complete lower lobe collapse normally pulls the major fissure downwards and medially, thus giving the collapsed lung a triangular shape. Lower lobe collapse because of diaphragmatic palsy usually results in a platelike atelectasis. Other shapes have been rarely described [4]. A segmental or sub-segmental collapse may be more subtle and can be completely missed on a chest X-ray. In the absence of major signs of fissure or mediastinal displacement, a collapse can sometimes be mistaken for other conditions, such as pneumonia.

When the lung remains collapsed for a long time, it loses its ability to expand because of several physiological processes like loss of surfactant, airway obstruction, interstitial edema, loss of lung elastic recoil, associated inflammatory changes, stagnation, and infection [5]. Although the time taken by such physiological changes is variable, it is generally believed that the lung may not expand if it has remained collapsed beyond six months.

## Case presentation

Presentation

A middle-aged lady in her forties presented in November 2023 with a history of exertional dyspnea and cough for three months. She is a known case of rheumatic heart disease (RHD) causing mitral valve regurgitation for many years. As she suffered from significant symptoms because of left ventricular failure, she underwent mitral valve replacement (MVR) surgery in 2016 using a tissue valve. This led to significant symptomatic recovery and helped her lead a normal life until her symptoms recurred in late year 2022. Investigations revealed that the mitral valve had degenerated and she underwent a repeat MVR surgery with a metallic valve in July 2023 that relieved her symptoms. A few weeks post-op, she again started developing frequent episodes of cough, dyspnea, and orthopnea. Three months post-op, she also suffered from atrial fibrillation, which required hospitalization and treatment with intravenous amiodarone. She was on oral anticoagulant therapy.

On examination, breath sounds were absent over the right lower thorax along with a dull note on percussion. She had significant orthopnoea and low peripheral oxygen saturation in the lying down position.

Investigations

**Figure 1:**
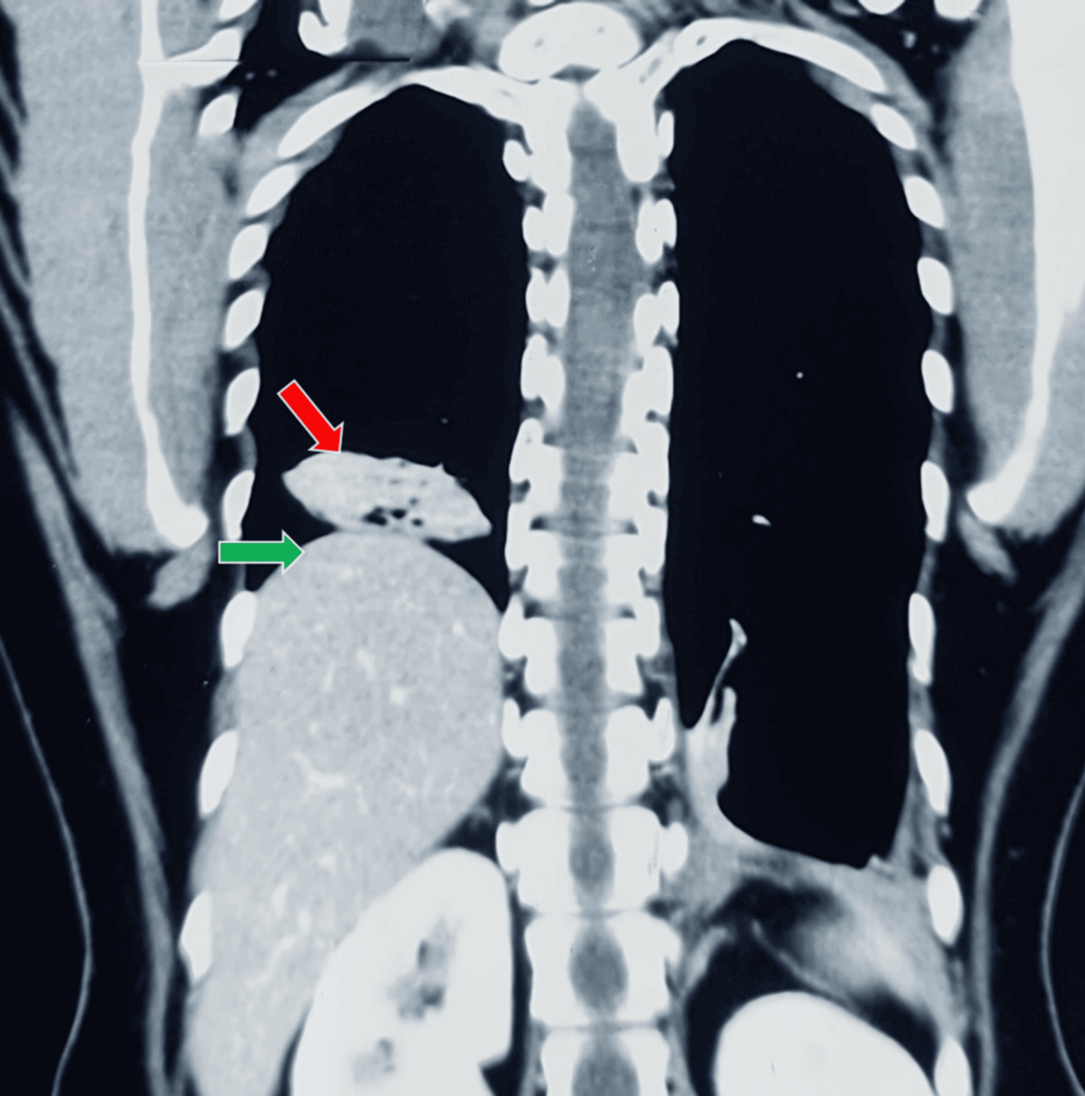
A CT scan of the chest showing an elevated right hemidiaphragm (green arrow), with a strange saucer-shaped lesion (red arrow) above it resembling an unidentified flying object (UFO).

A transthoracic echocardiography (2D-ECHO) showed an ejection fraction of 60% with no regional wall motion abnormalities and a mechanical prosthesis in situ in the mitral region that was functioning normally. A plain chest radiograph revealed an elevated right hemidiaphragm with a strange saucer (or disc-shaped) lesion above it, which was more evident on a coronal section of the CT scan, resembling an unidentified flying object (UFO) or a flying saucer, a term that was famous in the 1970s (Figure 1).

**Figure 2:**
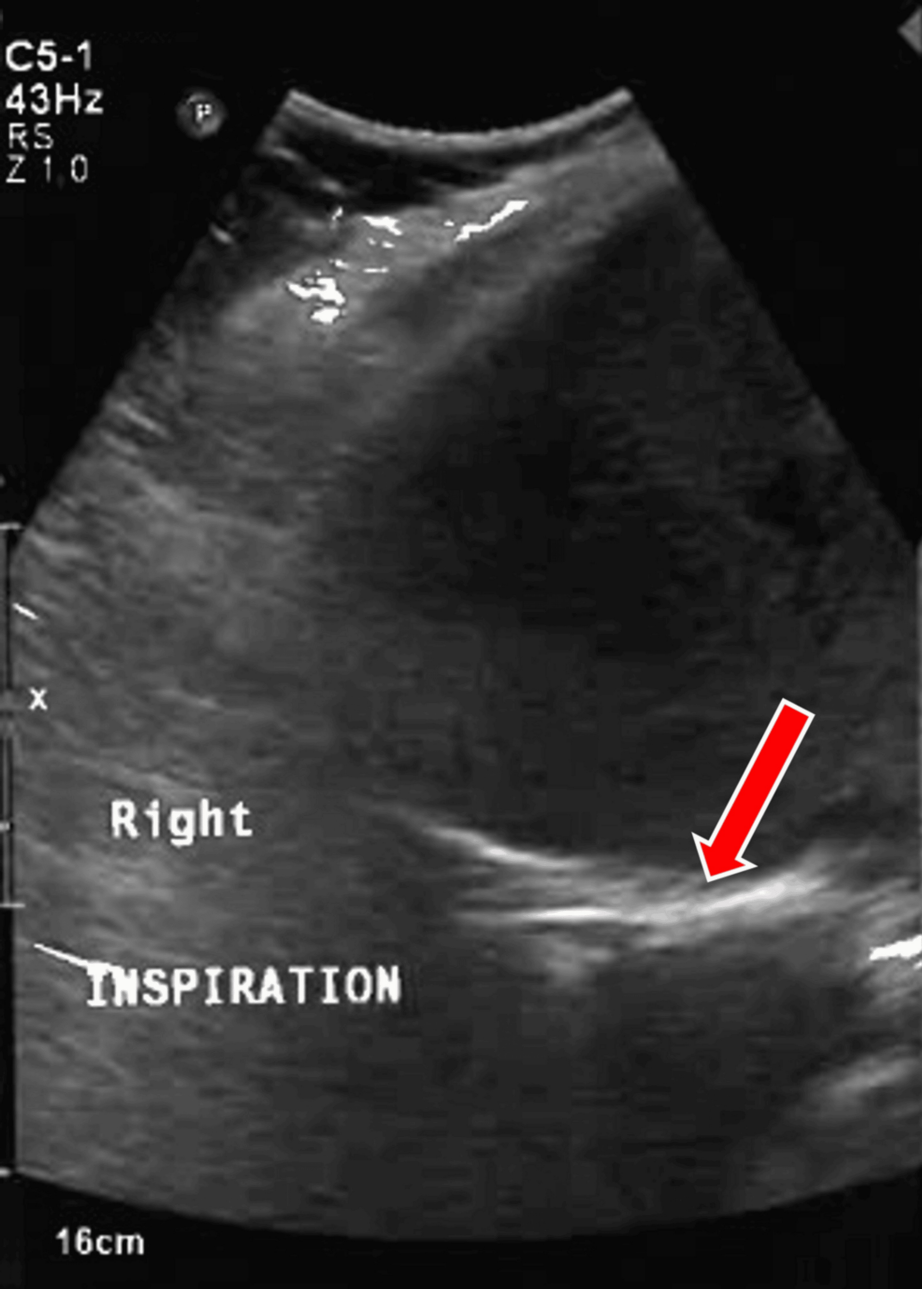
Ultrasonography of the abdomen and thorax portraying right diaphragmatic palsy. A red arrow denotes the diaphragm that shows no movement on inspiration.

**Figure 3:**
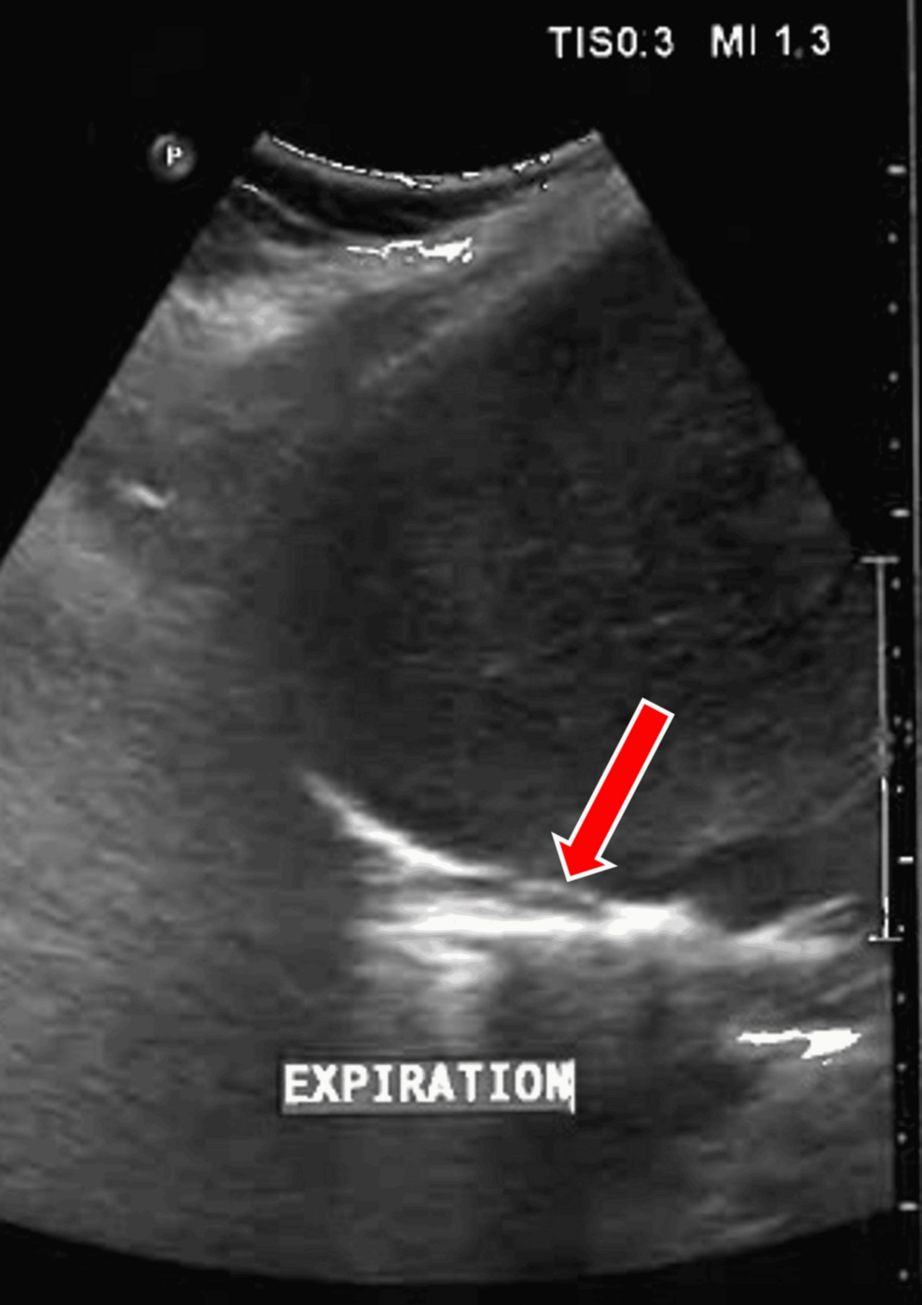
Ultrasonography of the abdomen and thorax portraying right diaphragmatic palsy. The red arrow denotes the diaphragm that shows no movement on expiration.

Ultrasonography of the abdomen on inspiration (Figure 2) and expiration (Figure 3) revealed right diaphragmatic palsy and also helped rule out right subpulmonic effusion.

**Figure 4:**
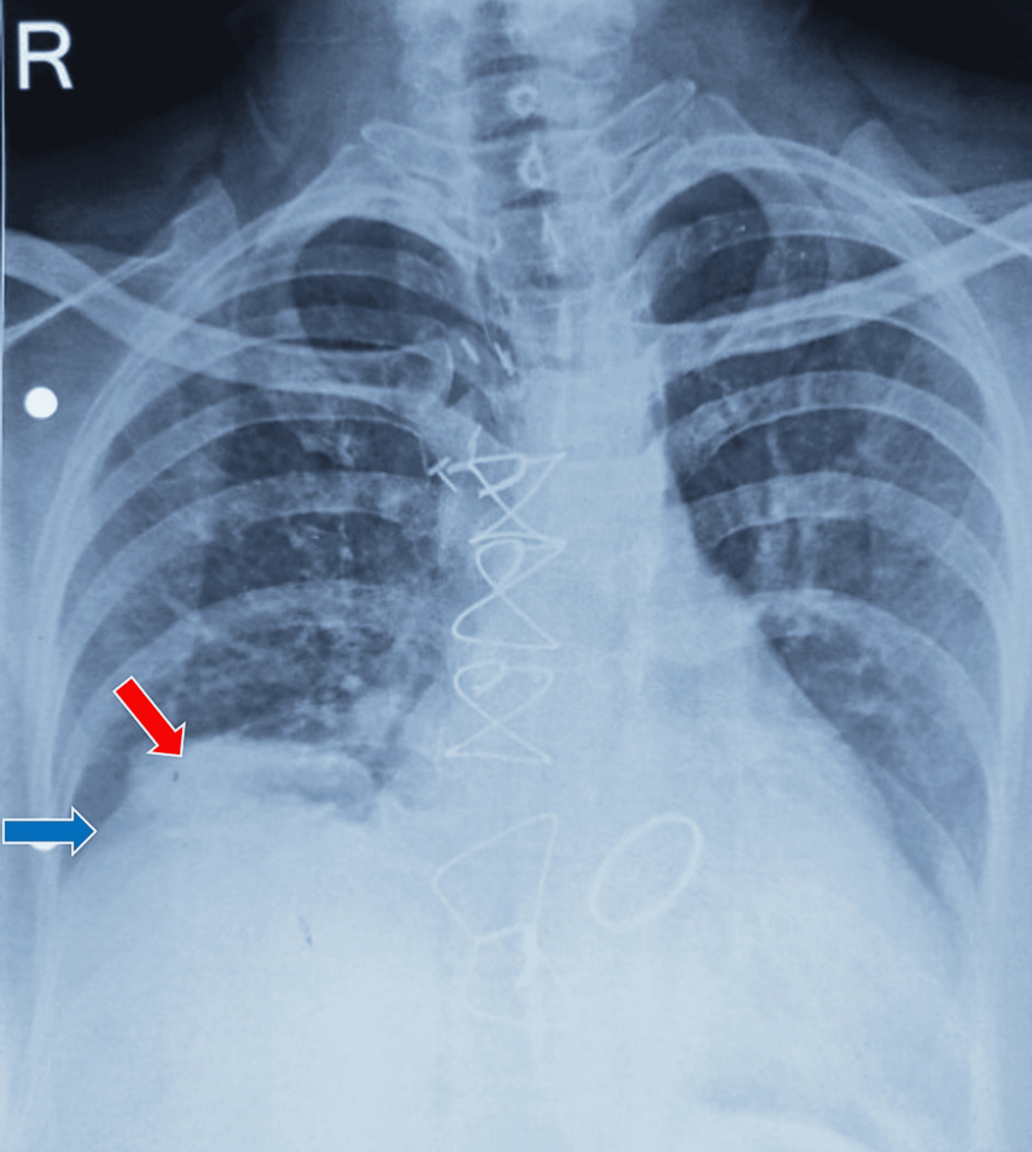
Chest radiograph showing the lesion (red arrow) and elevated right hemidiaphragm (blue arrow) post-redo mitral valve replacement surgery.

The possibility of lung sequestration, a congenital anomaly, was also initially thought of, but it was ruled out on examining her past chest X-rays serially as this lesion was not seen before the surgery. It was also realized that this lesion first appeared in the post-op period and had been gradually increasing in size and taking a strange saucer shape (Figure 4).

**Figure 5:**
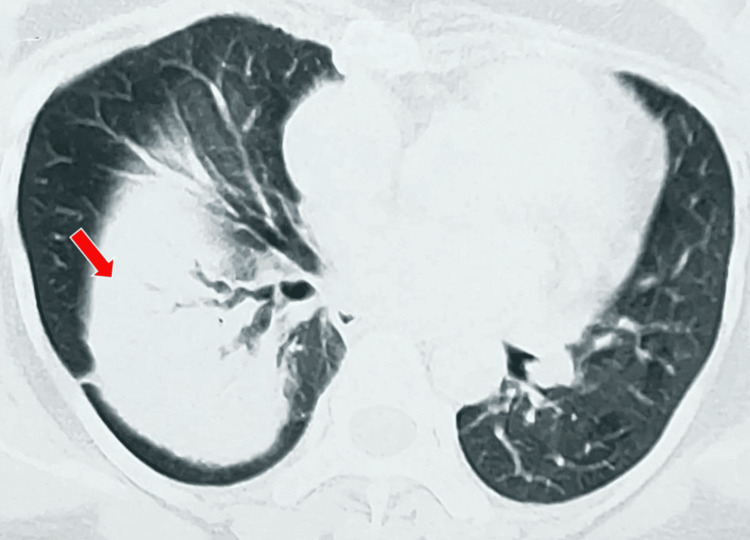
A CT scan of the chest showing soft tissue density (red arrow) in the right lower bronchus.

A high-resolution CT scan of the thorax showed that the lesion probably represented a collapsed lung silhouetting with the elevated right hemidiaphragm. The air bronchograms were tortuous, suggestive of early bronchiectasis. A soft tissue density was seen in the right lower bronchus, which needed further evaluation (Figure 5).

**Figure 6:**
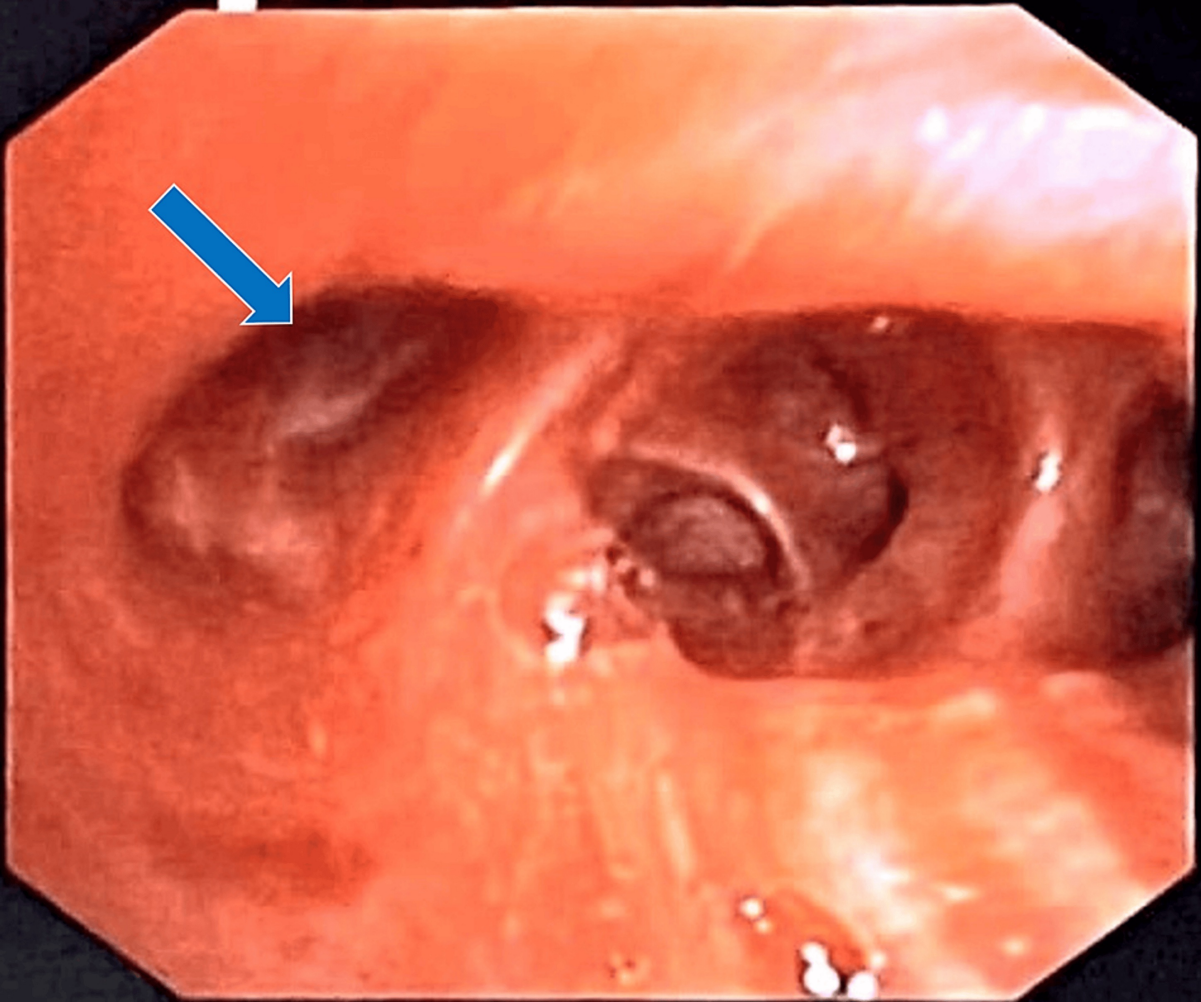
Image captured during fiberoptic bronchoscopy showing collapse of the airways (blue arrow).

A diagnostic fibreoptic bronchoscopy confirmed that all the segmental bronchi of the right lower lobe had collapsed because of external compression (Figure 6).

No intraluminal foreign body or any other obstructing pathology was found, thus suggesting that the intraluminal soft tissue shadow seen on the CT scan was probably just a mucus plug. To provide symptomatic relief from orthopnoea and prevent permanent damage to the right lower lobe, she was advised to undergo diaphragmatic plication.

Follow-up

**Figure 7:**
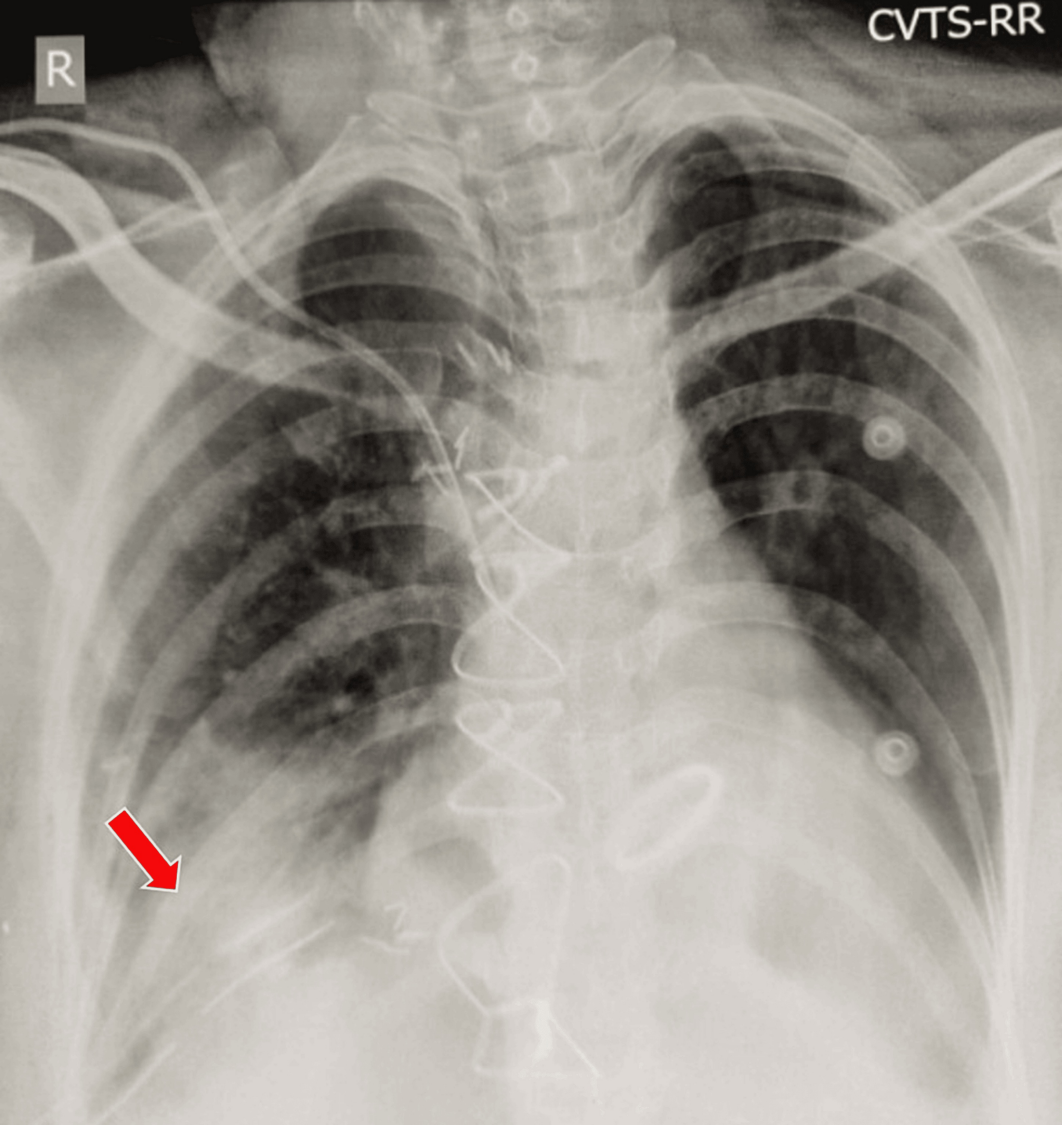
Chest radiograph immediately post-diaphragmatic plication showing no significant lesion resolution (red arrow).

**Figure 8:**
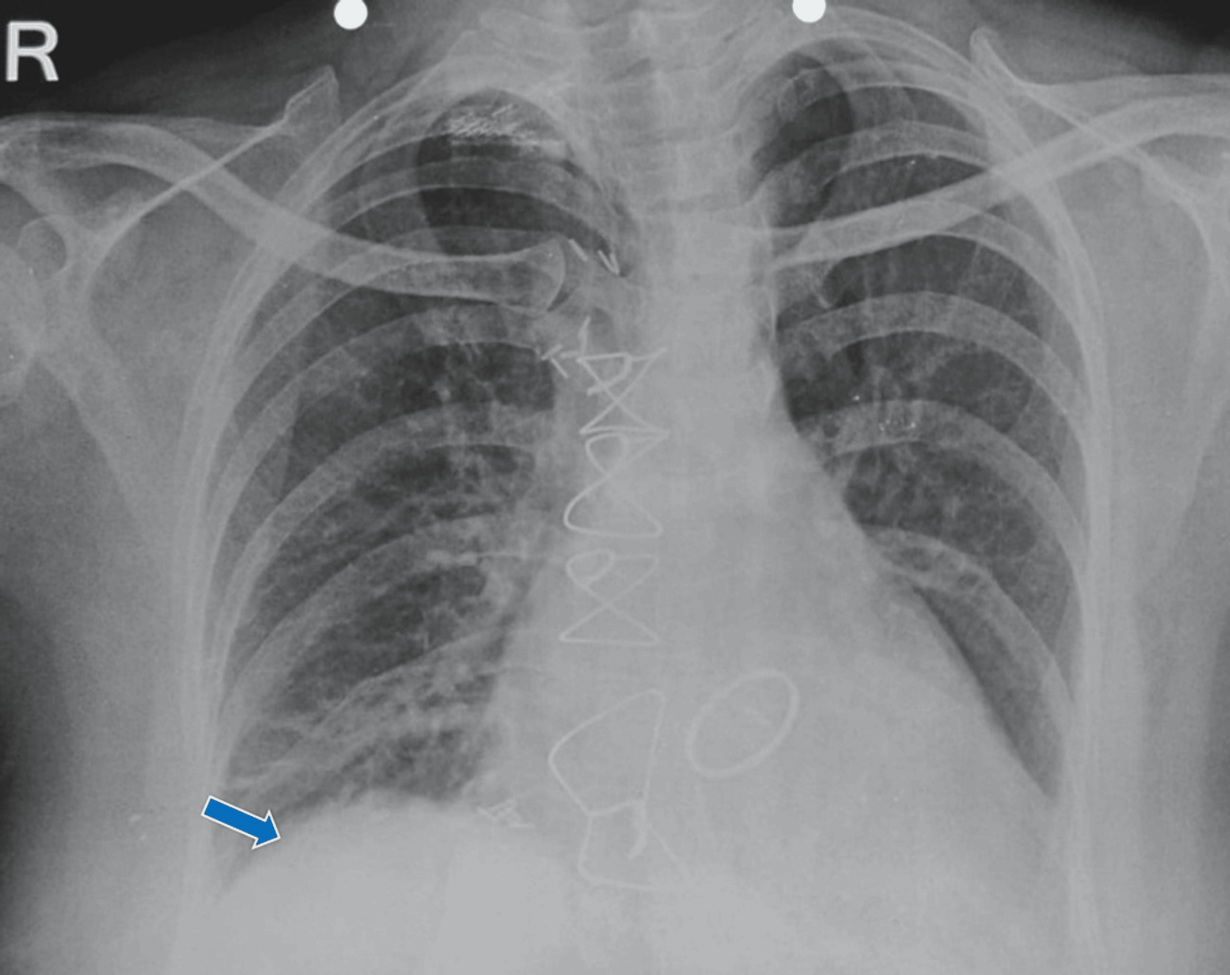
Chest radiograph showing near-complete resolution of the lesion (blue arrow) a month after diaphragmatic plication.

A diaphragmatic plication was performed in April 2024, which resulted in an immediate resolution of orthopnoea. Although the lower lobe remained collapsed immediately after the procedure (Figure 7), aggressive post-op chest physiotherapy and rehabilitation resulted in the complete expansion of the collapsed lung over one month (Figure 8). The lung lobe was thus successfully salvaged, and the patient was asymptomatic again.

## Discussion

Mitral valve replacement is generally advised when there is a significant leak causing left ventricular failure, conservative treatment seems inadequate for symptom control, or the patient is relatively young with a good life expectancy.

The choice between a tissue and a mechanical valve for mitral valve replacement depends on several factors, such as (a) patient age, wherein mechanical valves are preferred for younger patients because of their durability, while tissue valves are often chosen for older patients; (b) anticoagulation management, wherein mechanical valves require lifelong anticoagulation therapy while tissue valves typically need less aggressive anticoagulation; (c) lifestyle considerations, wherein tissue valves may be better for patients with high bleeding risk or difficulties with monitoring; (d) durability and longevity, wherein mechanical valves offer greater durability and are suitable for patients with a longer life expectancy; and (e) patient preference and overall health [6].

When a replaced mitral valve becomes dysfunctional, the initial assessment includes clinical evaluation and imaging, followed by optimization of medical therapy and anticoagulation. Repeat surgery may be needed for severe valve dysfunction, persistent symptoms, or complications such as endocarditis or thrombosis. Immediate postoperative failure is rare but can occur, with potential complications including bleeding, infection, thrombosis, and cardiac tamponade. Prevention involves meticulous surgical technique, careful monitoring, and appropriate management of anticoagulation and potential complications. Long-term management includes regular follow-up to address issues such as prosthetic valve degeneration and endocarditis [7].

Phrenic nerve injury is a recognized complication of thoracotomy, although its incidence is relatively low, generally reported to be between 0.5% and 2% of cases. In redo open heart surgeries, as in our case, the incidence will be much higher. This is because of dense adhesions of the heart to the pericardium, parietal, and visceral pleura [8]. Hence, the possibility of damage to the phrenic nerve during dissection increases. During sternotomy for MVR, an extra pericardial or intrapleural approach can be opted for as the heart is densely adherent to the pericardium.

Our patient underwent major surgery for the second time and had a recurrence of symptoms within a few weeks of the post-op recovery period. As the heart and prosthetic valve functions were assessed to be normal, she was referred to us for further evaluation.

An obvious abnormality noted on examination and on viewing the chest X-ray was right hemidiaphragm elevation, which would explain orthopnea. The strange saucer-shaped lesion seen above the hemidiaphragm was a diagnostic dilemma. It could have been a lung collapse because of diaphragmatic palsy or a subpulmonic pleural effusion. Vice versa, a collapsed lung pulling up the diaphragm was also a possibility. This needed further work-up. The shape of the lesion did not match the typical triangular-shaped collapse observed in most cases. A small possibility of sequestration was thought of as well, and there was also a possibility of pneumonia.

Lung sequestration is a congenital anomaly wherein, during embryological development, a part of the lung gets separated from the rest of the bronchial tree and derives its blood supply from the aorta. On viewing serial chest X-rays, it was clear that the lesion appeared after the second surgery and gradually increased over the next few months. It was not there pre-op, thus ruling out this differential diagnosis; thus, we could avoid further investigations.

Pneumonia is an acute infective process that would present with fever, purulent sputum, and leukocytosis, which was not the case. Tuberculosis was unlikely too, though not impossible.

A bronchoscopy was planned to eliminate the possibility of a foreign body in the right lower lobe causing its collapse, with post-collapse early distal bronchiectasis. Raised prothrombin time in this patient, who was on anti-coagulants, also proved to be a challenge as invasive procedures such as bronchoscopy, wherein manipulation might be required intra-procedure if a foreign body was found, made her a high-risk case [9]. On discussion with our cardiologist colleagues, we decided to switch the patient to subcutaneous enoxaparin injections, and oral anticoagulants were withheld to bring down the international normalized ratio (INR) for prothrombin time to normal. This approach would reduce the bleeding risk while adequately preventing cardiac thrombosis.

Fiberoptic bronchoscopy ruled out a foreign body and confirmed collapsing right lower lobe bronchi because of external compression.

A peripheral nerve injury, but with preserved structural continuity, may lead to demyelination or temporary dysfunction. In this condition, called neurapraxia, there is either a whole or partial loss of motor and sensory conduction. It usually shows recovery within three to six months [10]. Neuropraxia was ruled out in light of no improvement during the post-op period of more than six months.

Prolonged lung collapse leads to the loss of surfactant and the ability of the lung to inflate. Gradually, mucus stagnation and infection may set in, causing inflammation. The resultant fibrosis may lead to permanent damage to the lung, which loses its ability to expand forever.

In this case, one option was to offer continuous positive airway pressure (CPAP) to keep the lung inflated and wait for the diaphragmatic palsy to recover, which anyway, was a dim possibility. Moreover, prolonged chest physiotherapy and postural drainage would have been required to keep the lung free from stagnation or infection. Another treatment option was to do a nerve conduction study of the phrenic nerve and attempt phrenic nerve stimulation for prolonged periods to regenerate the nerve.

An easier and safer option was to plicate the hemidiaphragm. This would have immediate and permanent results. The treatment options were discussed with our colleagues from the Department of Cardiovascular Thoracic Surgery and also with the patient and her caretakers. The phrenic nerve can be repaired by nerve transfer and nerve graft techniques, along with diaphragmatic plication, but success in these cases has only been reported in limited cases [11]. Nerve repair techniques such as neurotization and neurolysis can be used in bilateral phrenic nerve palsy [12]. 

Counseling the patient involves explaining the condition, treatment options, and their implications. It was a tough decision for the patient considering that she already had a thoracotomy twice, and a repeat surgery would involve further morbidity. Undergoing another costly surgical procedure in the absence of medical insurance was a challenge too. Constant support from the medical team and perseverance in patient education and motivation were needed to overcome the challenges.

Diaphragmatic plication can be performed through thoracotomy or laparotomy. In this specific instance, a thoracotomy approach was chosen because of manageable lung adherence and separability from the diaphragm observed perioperatively. However, this procedure is not recommended for patients with dense adhesions and lower lobe collapse. For cases involving lung cancer primaries or metastases resulting in diaphragmatic palsy, laparotomy or laparoscopic approaches are preferred.

The most commonly utilized plication technique involves using a purse-string suture with number one prolene, which is often effective and comprehensive. Alternatively, mesh diaphragm plasty employs a prolene mesh to push the diaphragm downward into the abdomen. In cases of recurrence, a broader purse-string suture with prolene and multiple pledgets is employed.

The lung failed to expand immediately post-op. A multidisciplinary approach was needed, which involved aggressive chest physiotherapy, including techniques such as postural drainage to use gravity for mucus removal, percussion and vibration to loosen secretions, and breathing exercises to facilitate lung expansion. Incentive spirometry and coughing techniques were also used to improve lung function and clear mucus.

Salvaging the lung tissue and reestablishing good pulmonary function in this patient would not have been possible without consistent and persistent support from our multidisciplinary team, including pulmonologists, radiologists, cardiovascular thoracic surgeons, and physiotherapists. The coordinated and enthusiastic team effort bore fruit.

## Conclusions

While diverse shapes of the collapsed lung are possible, a disc-shaped lobar collapse resembling an unidentified flying object (UFO) is very rare and interesting. A systematic diagnostic approach helps in making a correct diagnosis. Various treatment options for diaphragm palsy have been discussed. The case also highlights the importance of patient education, rapport building, and a multidisciplinary approach for a favorable outcome.
